# Isolation of *Elizabethkingia anophelis* From COVID-19 Swab Kits

**DOI:** 10.3389/fmicb.2021.799150

**Published:** 2022-01-04

**Authors:** Liangcai Xu, Bo Peng, Yuxiang He, Yujun Cui, Qinghua Hu, Yarong Wu, Hongbiao Chen, Xiaofeng Zhou, Lili Chen, Min Jiang, Le Zuo, Qiongcheng Chen, Shuang Wu, Yang Liu, Yanming Qin, Xiaolu Shi

**Affiliations:** ^1^Department of Public Health Laboratory Sciences, School of Public Health, University of South China, Hengyang, China; ^2^Microbiology Lab, Shenzhen Center for Disease Control and Prevention, Shenzhen, China; ^3^State Key Laboratory of Pathogen and Biosecurity, Beijing Institute of Microbiology and Epidemiology, Beijing, China; ^4^Communicable Diseases Control and Prevention Division, Longhua District Center for Disease Control and Prevention, Shenzhen, China; ^5^Institute for Disinfection and Vector Prevention and Control, Shenzhen Center for Disease Control and Prevention, Shenzhen, China

**Keywords:** contamination, identification, multidrug resistance, heat tolerance, nosocomial infection

## Abstract

**Purpose:** To investigate and characterize the putative *Elizabethkingia anophelis* contaminant isolated from throat and anal swab samples of patients from three fever epidemic clusters, which were not COVID-19 related, in Shenzhen, China, during COVID-19 pandemic.

**Methods:** Bacteria were cultured from throat (*n* = 28) and anal (*n* = 3) swab samples from 28 fever adolescent patients. The isolated bacterial strains were identified using matrix-assisted laser desorption/ionization time of flight mass spectrometry (MALDI-TOF/MS) and the VITEK2 automated identification system. Nucleic acids were extracted from the patient samples (*n* = 31), unopened virus collection kits from the same manufacturer as the patient samples (*n* = 35, blank samples) and from unopened throat swab collection kits of two other manufacturers (*n* = 22, control samples). Metagenomic sequencing and quantitative real-time PCR (qPCR) detection were performed. Blood serum collected from patients (*n* = 13) was assessed for the presence of antibodies to *E. anophelis*. The genomic characteristics, antibiotic susceptibility, and heat resistance of *E. anophelis* isolates (*n* = 31) were analyzed.

**Results:** The isolates were identified by MALDI-TOF/MS and VITEK2 as *Elizabethkingia meningoseptica*. DNA sequence analysis confirmed isolates to be *E. anophelis*. The patients’ samples and blank samples were positive for *E. anophelis*. Control samples were negative for *E. anophelis*. The sera from a sub-sample of 13 patients were antibody-negative for isolated *E. anophelis*. Most of the isolates were highly homologous and carried multiple β-lactamase genes (*bla*_B_, *bla*_GOB_, and *bla*_CME_). The isolates displayed resistance to nitrofurans, penicillins, and most β-lactam drugs. The bacteria survived heating at 56°C for 30 min.

**Conclusion:** The unopened commercial virus collection kits from the same manufacturer as those used to swab patients were contaminated with *E. anophelis*. Patients were not infected with *E. anophelis* and the causative agent for the fevers remains unidentified. The relevant authorities were swiftly notified of this discovery and subsequent collection kits were not contaminated. DNA sequence-based techniques are the definitive method for *Elizabethkingia* species identification. The *E. anophelis* isolates were multidrug-resistant, with partial heat resistance, making them difficult to eradicate from contaminated surfaces. Such resistance indicates that more attention should be paid to disinfection protocols, especially in hospitals, to avoid outbreaks of *E. anophelis* infection.

## Introduction

Swab specimens are widely used for culturing respiratory pathogens (Miller et al., 2018). To facilitate the transportation and preservation of the microorganisms, freshly collected throat swabs are often stored and transported in collection tubes containing sterile preservation solution (transport medium) before laboratory testing. Transport medium is used to provide microorganisms with a suitable living environment where important requirements, such as pH and osmotic pressure, help maintain their viability. In China, during the COVID-19 pandemic, swab collection tubes were widely used as a SARS-CoV-2 prevention and control measure. From April to June 2020, three clustered fever outbreaks of unknown origin occurred in three different middle schools in Shenzhen, China. After screening for pathogens, qPCR identification found that the patients’ samples were negative for common respiratory pathogens, including SARS-CoV-2. However, we isolated *Elizabethkingia* bacteria from all patients’ samples, which are an emerging respiratory infection-causing pathogen, potentially leading to pneumonia in immunocompromised individuals (Hu et al., 2017; Juhász et al., 2018).

*Elizabethkingia*, a Gram-stain-negative bacillus, is aerobic, produces pale yellow colonies and can grow on MacConkey agar (Kim et al., 2005). In 1959, *Elizabethkingia* was discovered by a microbiologist at the United States Centers for Disease Control (CDC; King, 1959) and in 2011, Kämpfer et al. (2011) isolated *Elizabethkingia anophelis* from the midgut of *Anopheles gambiae*. In recent years, there have been increasing reports of *E. anophelis* infections threatening human lives, particularly in clinical settings (Teo et al., 2013; Lau et al., 2016; Perrin et al., 2017; Lin et al., 2018; Choi et al., 2019). In 2012, Teo et al. (2013) reported that an *E. anophelis* outbreak among five patients caused by nosocomial infections resulted in two deaths from sepsis. In 2016, in Hong Kong, China, Lau et al. (2016) reported 17 cases of *E. anophelis* infection, 12 of which were nosocomial. Another study found that the incidence of nosocomial infections caused by *E. anophelis* in South Korea during 2016–2017 was significantly higher than in previous years (Choi et al., 2019). A hospital in Taiwan reported 67 cases of *E. anophelis* infection in 12 years, of which 57 cases (85.1%) were hospital infections (Lin et al., 2018). In 2015 and 2016, the 63 *E. anophelis* infection cases reported in Wisconsin, United States, resulted in 19 deaths, and it is noteworthy that these were community-acquired infections, rather than hospital-acquired infections (Perrin et al., 2017).

The pathogenic mechanism and transmission route used by *E. anophelis* are not fully understood. The patients in the three fever clusters were all teenagers with normal levels of immunity. To explore whether the *Elizabethkingia* isolates were related to the fever outbreaks, we performed metagenomic analysis on the collected samples and investigated the phenotypic and molecular characteristics of the *Elizabethkingia* isolates.

## Materials and Methods

### Specimen Sources and Bacterial Isolation

In 2020, during the COVID-19 pandemic, adolescent patients presented with respiratory tract infection symptoms in three different fever clusters, where the first cases were detected on April 30th, May 7th, and June 3rd. Samples were collected from a total of 28 patients, using commercial disposable virus collection kits (manufacturer 1) to obtain throat (*n* = 28) and anal swabs (*n* = 3). In fever cluster 1, on April 30th, 15 throat swabs were collected (batch 1 of the virus collection kits), In fever cluster 2 (May 7th), six throat swabs and three anal swabs were collected using batch 2 of the virus collection kits, and during fever cluster 3 (June 3rd) seven throat swabs were taken using batch 3 of the virus collection kits (Supplementary Table S1). The collected samples were each stored in separate tubes containing Hank’s balanced salt solution, gentamicin, fungal antibiotics, calf serum albumin (fraction V), cryoprotectants, and biological buffers, and transported in a timely manner to the laboratory. Blood serum samples (*n* = 13) were collected on June 30. We collected 35 unopened virus collection kits from the batch 3 as blank samples. We also collected unopened virus collection kits from two other manufacturers as control samples, in the same period, with 13 samples from manufacturer 2 and nine samples from manufacturer 3. The details of all samples are shown in Supplementary Table S1. All patients in this study recovered within 1–3 days.

The collected patient samples were inoculated onto separate blood plates and cultured at 36°C. Single colonies were picked for identification 24 h later using gram staining microscopy and matrix-assisted laser desorption/ionization time of flight mass spectrometry (MALDI-TOF/MS; Bruker ultrafleXtreme, Karlsruhe, Germany). The VITEK2 Automated Identification System (BioMerieux, Lyon, France) was used with the gram-stain-negative biochemical card to investigate the biochemical reactions of the isolates and for positive identification.

### Microbial Metagenome Sequencing

Of the 31 patient samples, 24 (Fever cluster 1 and Fever cluster 2) were metagenomically sequenced, as well as five unopened virus collection kits (blank samples 1–5), which were used as controls. All patient samples (*n* = 31), 35 unopened virus collection kit samples (blank samples 1–35) and 22 unopened virus collection kit samples from two other manufacturers (control samples 1–22) were analyzed with qPCR to detect *E. anophelis*. From each sample tube, the transport medium was removed for whole nucleic acid extraction using a kit (Roche, MagNA Pure LC Total Nucleic Acid, Basel, Switzerland) and BGI (Shenzhen, China) conducted the metagenomic sequencing. The original metagenomic read data were further filtered using Trimmomatic (v0.39) for quality control. Taking the human genome sequence GRCh38 as a reference, Bowtie2^1^ was used to remove host reads. Metaphlan3^2^ was used to align metagenomic reads with the marker gene database (mpa_v30_CHOCOPhlAn) to identify the species distribution of the sample. The comparison database is unique clade-specific marker genes, which can accurately classify microorganism species. Visualization of the metagenomic analysis was achieved using Hcluts2^3^ and Graphlan.^4^

qPCR primers and probes were designed to detect *E. anophelis*. The target gene for detection encodes lipid A-disaccharide synthase (GenBank locus tag BD94_RS01570; Chew et al., 2018). The primers for the reaction were primer-F (5'-CGGAAGCAAGAAGTAGAA-3') and primer-R (5'-CCACATACTGCTCATAGAA-3'), used with the molecular beacon probe (5'-CGCAGGAGCACCTTCGTTGGCCTGCG-3'). The 5' end of the probe was connected to the Texas Red fluorescent group, and the 3' end of the probe was connected to the dabcyl quenching group. qPCR was performed in a 25-μl reaction volume containing 5 μl of sample DNA, 400 nM of forward and reverse primers, 250 nM of probe and 1× Pro Taq HS Premix Probe from the qPCR Kit (Accurate Biology, Hunan, China) on an Applied Biosystems 7500 real-time PCR instrument (Waltham, MA, United States). The initial activation temperature (95°C, 5 min) was followed by 40 cycles of 95°C for 15 s and 55°C for 45 s. We tested 31 patient samples, blank samples 1–35 and control samples 1–22 using this method.

### Patient-Specific Antibody Detection

Enzyme-linked immunosorbent assays (ELISA) was performed on blood sera of a sub-sample of patients (*n* = 13; Supplementary Table S1) to determine whether the patients had antibodies to the isolated *E. anophelis*. Using the indirect method, the isolated whole cells of *E. anophelis* were used as the antigen. This involved coating 96-well plates with the whole bacterial antigen on the carrier. An ELISA coating buffer (Sangon Biotech, Shanghai, China) was used to dilute the whole bacterial cell antigen to 10 ng/μl, and 100 μl of diluted antigen was added to the 96-well plate (carrier), which was incubated at 37°C for 1 h. The liquid was then discarded and 150 μl of skimmed milk powder (5%) was added to each well. Plates were then kept at 37°C for 1 h. Using skimmed milk powder (2%) as the diluent, the patient’s serum was diluted in multiples (range 1:2–1:2,048), and a 100 μl dilution series was added to each test well, and incubated at 37°C for 1.5 h. After washing three times with ELISA Washing Buffer (Sangon Biotech), 100 μl of goat anti-human IgG secondary antibody (horseradish peroxidase-labeled) was added and incubated at 37°C for 40 min. After washing three times with ELISA Washing Buffer, 100 μl ELISA Chromogen Solution (Sangon Biotech) was added and incubated at 37°C for 3 min. Finally, 50 μl of ELISA Stopping Solution (Sangon Biotech) was added. The optical density (OD) value was read using a microplate reader (KHB, Shanghai, China). The one-sided 99% CI of the negative control OD value was taken as the cut-off value and the following formula was applied: $\bar{X}\;$ + 2.56S ( $\bar{X}\;$ : the mean OD value of the negative control; S: the SD of the negative control’s OD value). Immunized mouse serum was injected subcutaneously with *E. anophelis* as a positive quality control material, and labeled goat anti-mouse antibody used as a secondary antibody. For the negative control, healthy human serum (serum collected by the disease surveillance system) was used.

### Whole Genome Sequencing Analysis

Nucleic acids were extracted from 31 isolates using a commercial kit (TIANGEN, Beijing, China). Whole genome sequencing and its preliminary evaluation and filtering were performed on the Illumina platform. The original data were further filtered using Trimmomatic (v0.39) for quality control to obtain valid data. *De novo* genome assembly used the SPAdes gene assembly software (V3.9.1). Core-genome single-nucleotide polymorphisms (core-SNPs) were identified using the Snippy pipeline v4.3.8.^5^ The maximum likelihood trees were constructed based on the alignment of core-SNPs using RAxML (v8.2.12) under the GTRGAMMAX model with 1,000 bootstrap iterations. Carriage of antibiotic resistance and virulence genes was assessed using ABRicate (v 0.8). The vfdb^6^ and ResFinder^7^ databases were used as references at a cut-off of 75% sequence identity and 75% sequence coverage from draft genome assemblies.

### Antibiotic Susceptibility Testing

A drug susceptibility board (GN4F; Thermo Fisher Scientific, Waltham, MA, United States) was used to study the MIC levels of 31 isolates against 24 antibiotics and antibacterial agents: piperacillin/tazobactam, ticarcillin/clavulanic acid, trimethoprim/sulfamethoxazole, ampicillin/sulbactam, imipenem, ampicillin, piperacillin, meropenem, ceftazidime, aztreonam, cefepime, ceftriaxone, doripenem, ertapenem, cefazolin, amikacin, gentamicin, tobramycin, tetracycline, minocycline, tigecycline, levofloxacin, ciprofloxacin, and nitrofurantoin. *Escherichia coli* ATCC 25922 was used as the quality control strain. We referred the drug susceptibility breakpoints from the US Food and Drug Administration (FDA) for non-Enterobacteriaceae to interpret the results for ampicillin/sulbactam and from Clinical and Laboratory Standards Institute (CLSI) for non-Enterobacteriaceae to interpret the results for the remaining 17 antibiotics.

### Heat Resistance Tests

Considering the biological safety of the laboratory, the collected patient specimens were heated and sterilized at 56°C, but we were able to separate *E. anophelis* from these samples. Therefore, we conducted heat resistance tests on the isolates (*n* = 31). Separate bacterial suspensions, each with 0.5 McKenzie turbidity in physiological saline, were prepared and heated with a metal heater set at 37, 56, 66, and 76°C each for 10, 20, and 30 min. Each treated bacterial suspension was removed, inoculated onto a blood agar plate, cultured at 37°C for 24 h and bacterial growth was observed.

## Results

### Pathogen Screening Results

**Figure 1:**
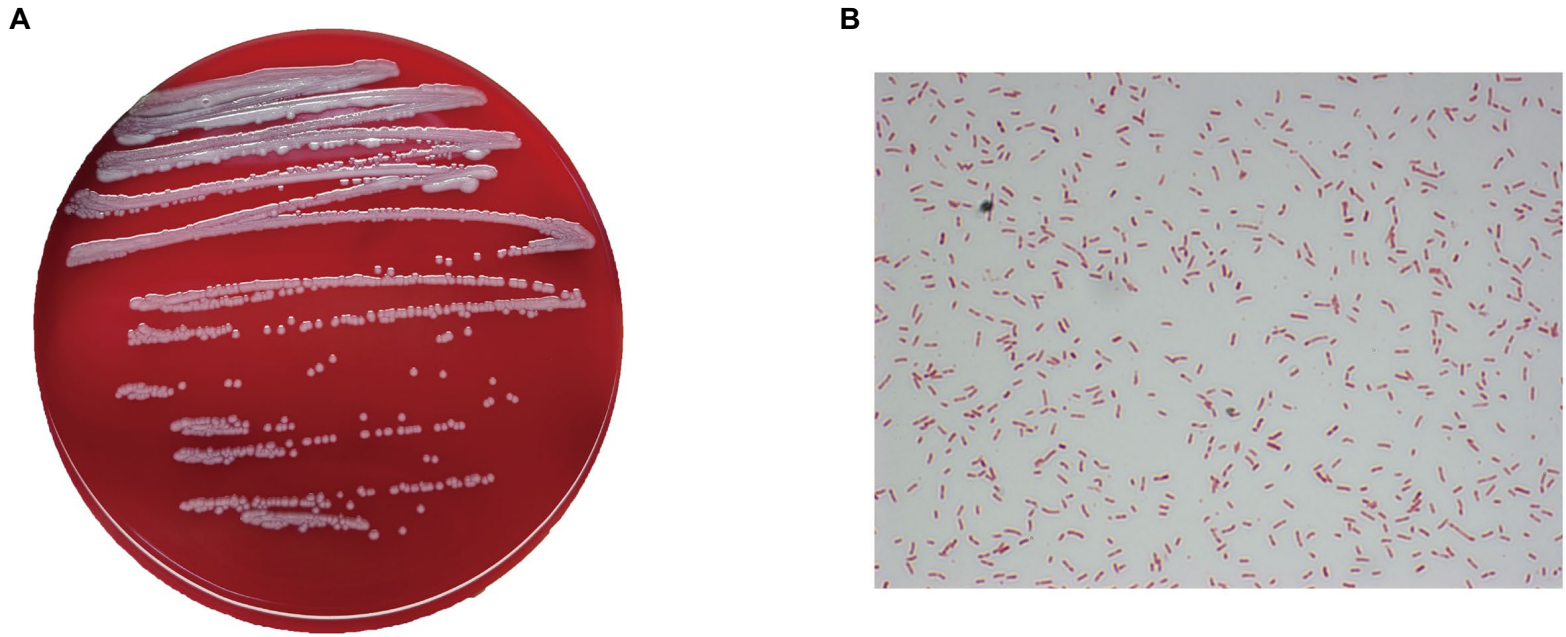
Phenotypic characteristics of the isolates. **(A)** Growth of one of the isolates on a blood agar plate. **(B)** Gram staining shows the colonies to be Gram-stain-negative bacilli (×1,000).

After isolation, all 31 patient samples were cultured, and bacterial colony phenotypes were all consistent. The colony of the bacteria after isolation and culture is shown in Figure 1A. The colony surfaces were smooth and light-yellow colored. In all samples, the gram-staining of the colonies confirmed the isolate to be a Gram-stain-negative bacillus (Figure 1B) and the MALDI-TOF/MS analysis results indicated colonies to all be *Elizabethkingia meningoseptica*, which was further shown by the VITEK 2 system. In addition, *Enterococcus faecalis* was also isolated in a small part of the samples (4/31). The test results for 47 biochemical indicators suggesting *E. meningoseptica* are shown in Supplementary Table S2. In six experiments, except for the differences in the biochemical reactions of D-mannose (dMNE), lipase (LIP), and beta-galactosidase (BGAL), the profiles of the other 44 biochemical indicators did not differ.

### Microbial Analysis

**Figure 2:**
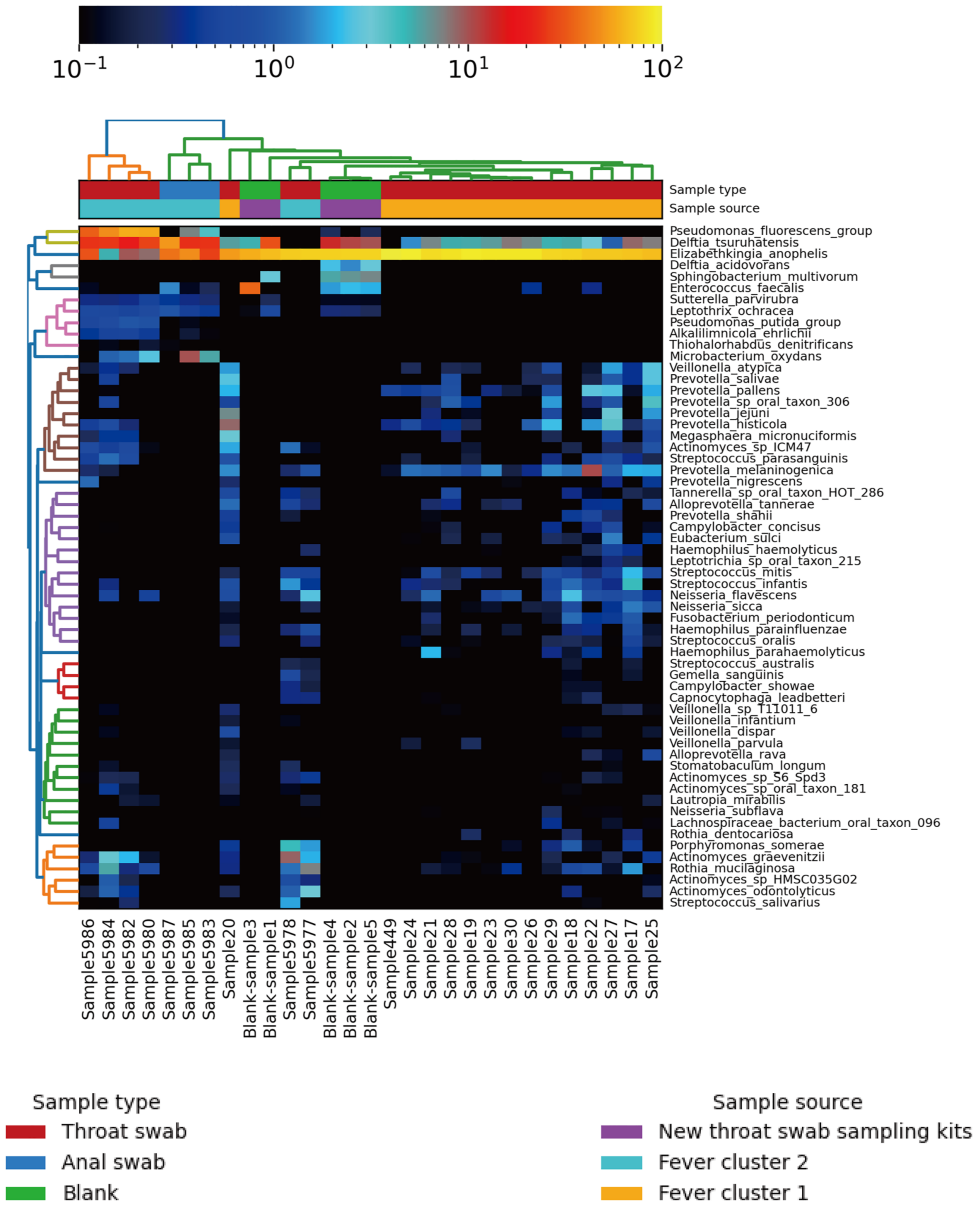
Heat map of population distribution of the 60 most abundant bacterial species in the 24 patient specimens and five new (unopened) virus collection kits. The top tree map is clustered based on the species structure and relative abundance in the samples. After cluster analysis, the sample is mainly divided into three clusters.

**Figure 3:**
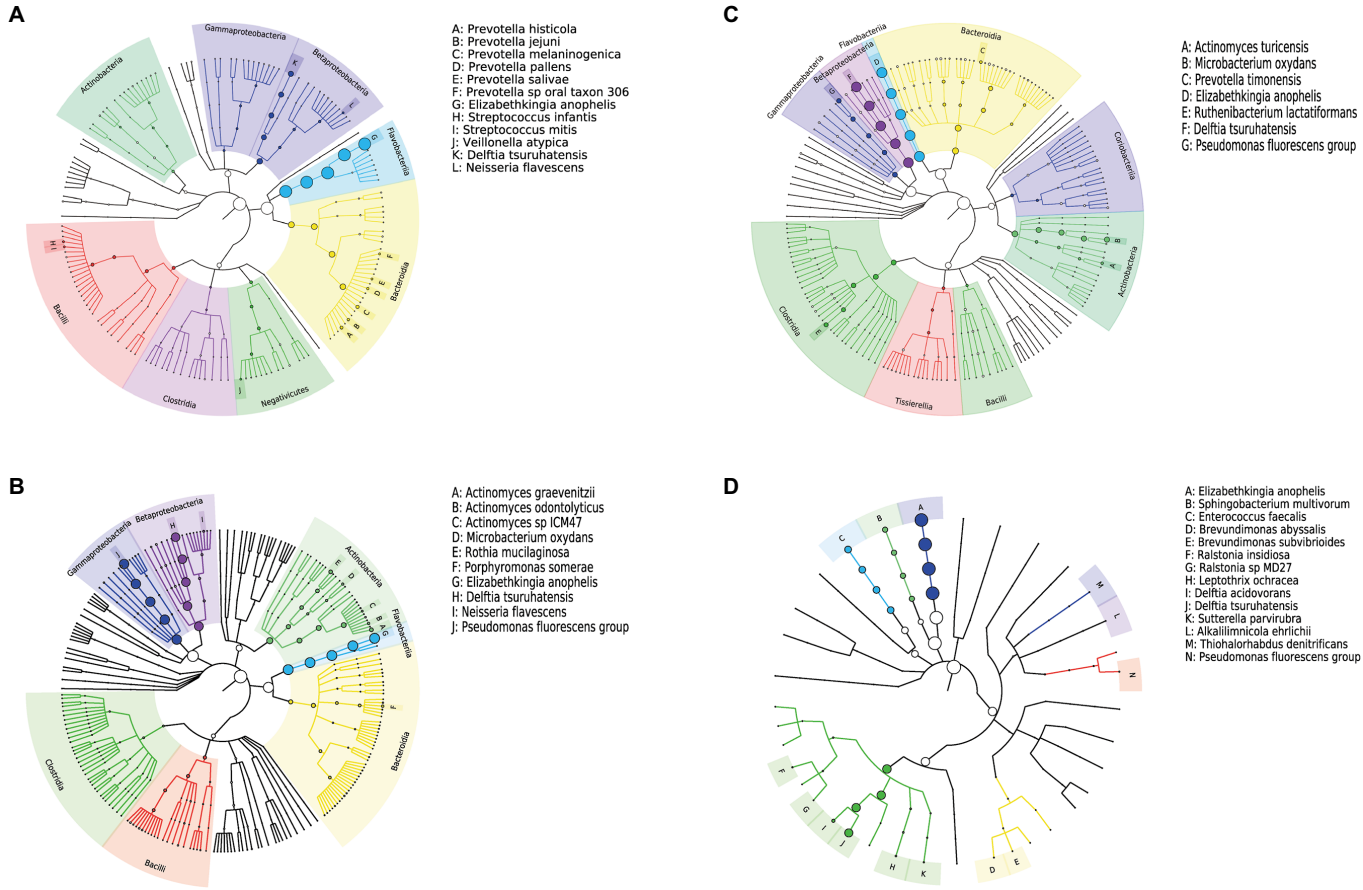
Phylogenetic tree of the microbiome of different samples; **(A)** 15 throat swab samples in Fever cluster 1; **(B)** six throat swab samples in Fever cluster 2; **(C)** three anal swab samples in Fever cluster 2; and **(D)** five blank samples. The nodes from inside to outside each tree represent the world, phylum, class, order, family, genus, and species, and the size of the circle at the node represents abundance.

All samples contained only bacteria, no viruses, and were divided into three clusters according to the microbial diversity and relative abundance in the samples. All patients and blank samples were positive for *Elizabethkingia*, with high abundance levels (Figure 2). In contrast to the MALDI-TOF/MS and VITEK2 results, the metagenomic sequence alignments showed that the isolated bacterium was *E. anophelis*. All patient samples from Fever cluster 1 were throat swab samples, and the bacterial species structure was concentrated in one cluster, mainly containing *E. anophelis* and *Delftia tsuruhatensis* (Figure 3A). Fever cluster 2 patient samples, including both throat and anal swab samples, contained bacterial species structures distributed across the three clusters. In Fever cluster 2 patient samples, the throat swab samples (*n* = 6) mainly contained *E. anophelis*, *Pseudomonas fluorescens*, and *D. tsuruhatensis* (Figure 3B), and anal swab samples (*n* = 3) mainly contained *E. anophelis* and *D. tsuruhatensis* (Figure 3C). The blank samples 1–5 were contaminated with numerous bacterial types, mainly *E. anophelis*, *D. tsuruhatensis*, *Sphingobacterium multivorum*, and *Enterococcus faecalis* (Figure 3D). The qPCR results showed that all 31 patient samples and the 35 unopened throat swab collection kits (blank sample 1–35) were positive for *E. anophelis*, but 22 control samples (control sample 1–22) were negative for *E. anophelis*. In the ELISA tests, serum samples from 13 patients did not react with the isolated *E. anophelis*, suggesting that the *E. anophelis* isolated from patient swab samples did not originate from the patients. The pathogen responsible for patients’ respiratory symptoms was not *E. anophelis*; rather the throat swab collection devices were contaminated with this bacterium. In Fever cluster 1, all patient samples contained *Haemophilus parainfluenzae*, but the relative abundance was low. No suspected co-infecting pathogens were found in the Fever cluster 2 and Fever cluster 3 patient samples.

### Genomic Analysis

**Figure 4:**
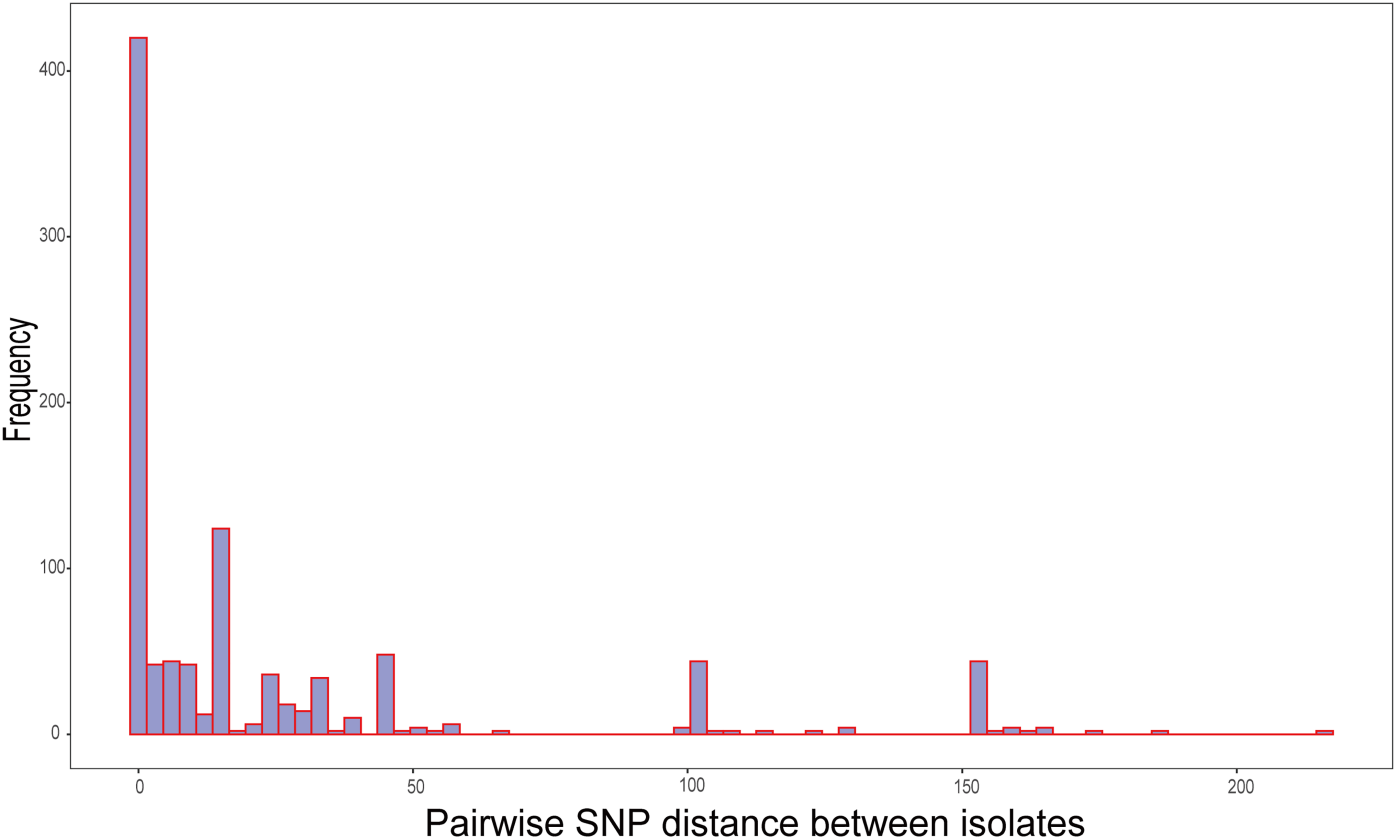
Distribution of pairwise single-nucleotide polymorphism (SNP) distances between all isolates.

**Figure 5:**
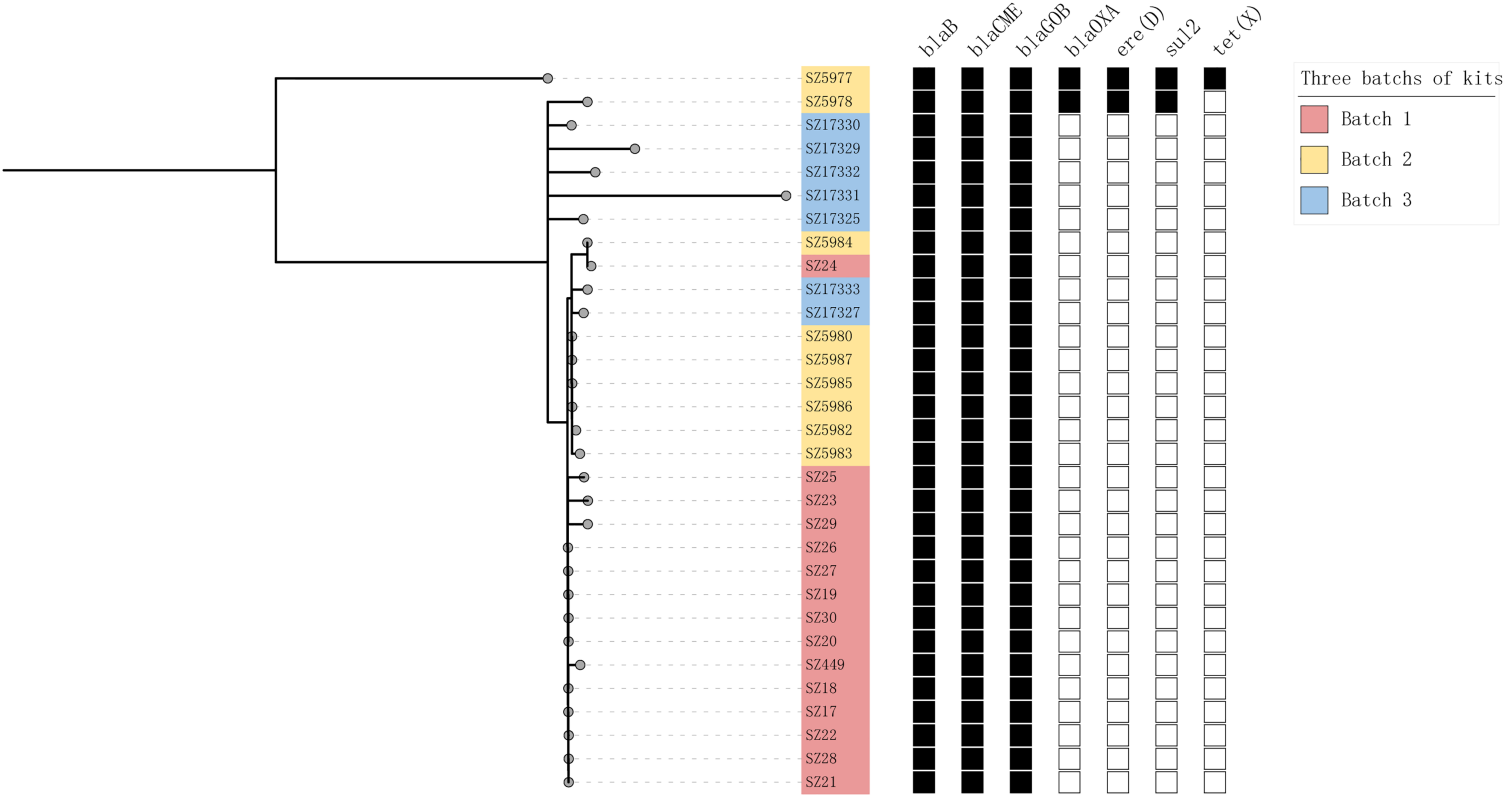
Maximum likelihood trees for all 31 isolates. Pink, yellow, and blue represent different sources of isolates. The black boxes represent the presence of the corresponding gene, and the blank boxes represent the absence of these genes.

The genetic distances between the 31 isolates were relatively close. The pairwise SNP distances of most isolates were between 0 and 19, and the largest SNP distance was 204 between SZ5977 and SZ17331 (Figure 4). The isolates from samples derived from batch 1 of the swab kits, and some from batch 2, were highly homologous, and the SNP distance between the isolates was ≤10. All the isolates carried the β-lactamase genes *bla*_B_, *bla*_CME_, and *bla*_GOB_. In addition, isolates SZ5977 and SZ5978 carried the β-lactamase gene *bla*_OXA_, the anti-macrolide gene *ere(D)* and the sulfa-resistant gene *sul2*. Only SZ5977 carried the anti-tetracycline gene *tet(X)* (Figure 5). No virulence gene was found in any isolate.

### Antimicrobial Susceptibility Patterns

The antimicrobial susceptibility of all the bacterial strains was highly consistent (Table 1). The isolates displayed a high resistance rate to ticarcillin/clavulanic acid (29/31), trimethoprim/sulfamethoxazole (28/31), ampicillin/sulbactam (31/31), ceftazidime (31/31), cefepime (29/31), ceftriaxone (31/31), imipenem (31/31), meropenem (31/31), aztreonam (31/31), gentamicin (21/31), tobramycin (31/31), and tetracycline (31/31). The isolates displayed high MIC levels to doripenem, ertapenem, cefazolin, nitrofurantoin, and ampicillin (Table 1). Bacteria were susceptible to piperacillin/tazobactam (31/31), amikacin (27/31), minocycline (31/31), levofloxacin (29/31), and ciprofloxacin (17/31), and displayed a low MIC level to tigecycline. Except for their susceptibility to piperacillin/tazobactam and an interpretation of piperacillin, the isolates were completely resistant to the other 12 β-lactam antibiotics. These isolates were generally highly resistant to β-lactam antibiotics and to nitrofuran (nitrofurantoin) and penicillin (ampicillin).

**Table 1:** Antimicrobial susceptibility profiles of the *Elizabethkingia anophelis* isolates, as determined by the broth microdilution method.

	Antimicrobial agents	Breakpoint (mg/L)			MIC range (mg/L)	Number of isolates		
		S	I	R		S	I	R
β-Lactam combination agents	Piperacillin/tazobactam constant	≤16/4	32/4–64/4	≥128/4	≤8/4–16/4	31	0	0
	Ticarcillin/clavulanic acid	≤16/2	32/2–64/2	≥128/2	≤8/2– > 64/2	2	0	29
	Trimethoprim/sulfamethoxazole	≤2/38	-	≥4/76	≤2/38– > 4/76	3	0	28
	Ampicillin/sulbactam	≤8/4	16/8	≥32/16	>16/8	0	0	31
Cephems	Ceftazidime	≤8	16	≥32	>16	0	0	31
	Cefepime	≤8	16	≥32	16– > 32	0	2	29
	Ceftriaxone	≤8	16–32	≥64	32– > 32	0	0	31
	Cefazolin	-	-	-	>16	-	-	-
Carbapenems	Imipenem	≤4	8	≥16	>8	0	0	31
	Meropenem	≤4	8	≥16	>8	0	0	31
	Doripenem	-	-	-	>4	-	-	-
	Ertapenem	-	-	-	8– > 8	-	-	-
Penicillins	Aztreonam	≤8	16	≥32	>16	0	0	31
	Piperacillin	≤16	32–64	≥128	≤16–64	1	30	0
	Ampicillin	-	-	-	>16	-	-	-
Aminoglycosides	Amikacin	≤16	32	≥64	16–32	27	4	0
	Gentamicin	≤4	8	≥16	8– > 8	0	10	21
	Tobramycin	≤4	8	≥16	>8	0	0	31
Tetracyclines	Tetracycline	≤4	8	≥16	>8	0	0	31
	Minocycline	≤4	8	≥16	≤1	31	0	0
	Tigecycline	-	-	-	≤1–8	-	-	-
Quinolones	Levofloxacin	≤2	4	≥8	≤1–4	29	2	0
	Ciprofloxacin	≤1	2	≥4	1– > 2	17	12	2
Nitrofurans	Nitrofurantoin	-	-	-	>64	-	-	-

### Heat Resistance

Of the 31 isolated *E. anophelis* strains, eight, four, and eight strains grew in cultures after being heated to 56°C for 10, 20, or 30 min, respectively. A temperature greater than 66°C for more than 10 min killed all isolates.

## Discussion

Throat swabs are widely used to collect clinical samples for respiratory disease pathogen detection. It is therefore essential that commercial swab collection kits are sterile, both for analysis accuracy and to ensure pathogens are not transmitted to patients. We isolated highly homologous strains of *E. anophelis* from the transport medium of three batches of commercial virus collection kits, suggesting that the transport medium was contaminated before it was aliquoted. Once contaminated, the transport medium, designed to maintain microorganisms, likely provided a suitable living environment for bacteria to grow and multiply, though bacterial growth was not observed upon visible inspection. Bacterial contamination of swab transport medium may affect the quality of preservation and RNA extraction, leading to the risk of either false positive or negative detection of SARS-CoV-2 (Rahbari et al., 2021). The relevant manufacturer was informed of the contamination, though no response was received as to the possible cause. In subsequent production, to ensure continued and effective SARS-CoV-2 testing, the manufacturer conducted strict disinfection and sterilization of the production workshop to reduce the risk of bacterial contamination. *E. anophelis* has not been detected in their latest batch of unopened virus collection kits. Although *Haemophilus parainfluenzae* was detected in patient specimens of fever cluster 1, it was not isolated and the causative agent of the three fever outbreaks was not identified.

In 2018, based on genome-wide comparisons, the *Elizabethkingia* genus was divided into six species, including *E. meningoseptica*, *E. anophelis*, *Elizabethkingia miricola*, *Elizabethkingia bruuniana*, *Elizabethkingia ursingii*, and *Elizabethkingia occulta* (Doijad et al., 2016; Nicholson et al., 2018). Identifying *Elizabethkingia* to the species level is challenging; however, as traditional identification methods (e.g., biochemical identification and MALDI-TOF/MS) cannot reliably be used. Using the MALDI-TOF/MS and VITEK2 systems, the strains we isolated were typed as *E. meningoseptica*; however, the higher resolution metagenomic analysis identified them as *E. anophelis*, and is undoubtedly the most accurate. Commercial identification systems based on biochemical phenotyping and MALDI-TOF/MS are limited by the lack of reference database information on all *Elizabethkingia* species, and are therefore unsuitable for correctly identifying *Elizabethkingia* to the species level (Arbune et al., 2018; Govindaswamy et al., 2018; Lin et al., 2019; Malik and McLeod, 2019). At present, the latest MicrobeNet MALDI-TOF/MS database,^8^ developed by Bruker and United States CDC, contains three *Elizabethkingia* species, including *E. meningoseptica*, *E anophelis*, and *E. miricola*. This provides the latest reference for the species identification of *Elizabethkingia*, but it still lacks the additional three *Elizabethkingia* species. The biochemical test results on the isolated *E. anophelis* provide a reference point for future biochemical identification. It has been shown that specific gene fragments can be PCR amplified to distinguish *E. anophelis* and *E. meningoseptica* (Holmes et al., 2013; Chew et al., 2018). The primers and probes designed in the present study detect *E. anophelis* specifically, and can therefore be used for reference in future studies.

*Elizabethkingia* has previously been shown to have strong and broad-range antibiotic resistance (Han et al., 2017; Govindaswamy et al., 2018; Wang et al., 2019, 2020), though there is no currently established criterion for the antibiotic susceptibility breakpoint. Infections by multidrug-resistant bacteria have brought a huge burden of disease to society (Zhen et al., 2019). Consistent with previous findings, the strains isolated in this study displayed broad-spectrum antibiotic resistance, especially to the β-lactam antibiotics associated with the β-lactamases (*bla*_B_, *bla*_GOB_, and *bla*_CME_) carried by the isolates. The isolates have high homology, so their antibiotic susceptibility profiles are extremely similar. The isolates were most susceptible to piperacillin/tazobactam, minocycline, and quinolones, in line with the 51 strains of *E. anophelis* collected in South Korea (Han et al., 2017), and provide a reference basis for clinical medication. In the context of the COVID-19 pandemic, for biosafety, the specimens that we analyzed were heated to 56°C before separation. Subsequent *E. anophelis* isolation confirmed its relative heat-resistance, also providing reference for the prevention and control of hospital infections and disinfection practices.

In recent years, there have been many reports of *Elizabethkingia* infections, most of which are sporadic infections in immunocompromised individuals, and only a few caused infection outbreaks (Teo et al., 2013; Moore et al., 2016; Perrin et al., 2017). The outbreak in Wisconsin, United States, is currently the only known community-related infection outbreak, though the source of the outbreak has not been identified despite exploring health products, personal care products, food, tap water, and human-to-human transmission as potential sources (Perrin et al., 2017). Elsewhere, *Elizabethkingia* has been isolated from a hospital environment and water source (Kyritsi et al., 2018), and it has been reported that *E. anophelis* can be indirectly transmitted to patients by the hands of medical staff (Yung et al., 2018). Nosocomial infections caused by antibiotic-resistant bacteria, such as *E. anophelis*, can have a high mortality rate and pose a serious threat to public health (Xia et al., 2016). Methicillin-resistant *Staphylococcus aureus* (MRSA), for example, is a primary pathogen of nosocomial infections (Xia et al., 2016) and has shown an increased rate of community infections in recent years (Otto, 2013; Monaco et al., 2017). *Elizabethkingia anophelis* has high antibiotic and thermal resistance, and has caused both nosocomial and community infections, suggesting cause for concern, particularly for immunocompromised patients. Strengthening prevention and control of *E. anophelis* infections is essential.

## Conclusion

We isolated *E. anophelis* from throat and anal swab samples collected from patients involved in three fever epidemic clusters during the COVID-19 pandemic. *Elizabethkingia anophelis*, however, was not the pathogenic agent of these clusters, but rather a contaminant in a commercial virus sampling kit transport medium. The causes of the three fever outbreaks in this study remain unidentified. As all swab sampling kits provided by the manufacturer, from three batches, were contaminated with *E. anophelis*, the contamination source is likely the same. This study highlights that *Elizabethkingia* should be identified to the species level using DNA sequence-based techniques. The *E. anophelis* isolated in this study has strong resistance to β-lactam drugs, nitrofurans, penicillin, and some aminoglycoside antibiotics. It is notable that the isolated bacterium also has a degree of heat resistance and is therefore not easy to kill, posing a significant infection risk to susceptible people. The high resistance of *E. anophelis* is therefore cause for concern, especially in the hospital environment. More research is needed on the characteristics of *E. anophelis* to ensure it can be effectively eliminated and to prevent devastating hospital outbreaks, similar to those of MRSA.

## Data Availability Statement

The datasets presented in this study can be found in online repositories. The names of the repository/repositories and accession number(s) can be found at: https://www.ncbi.nlm.nih.gov/genbank/, PRJNA764017, https://www.ncbi.nlm.nih.gov/genbank/, PRJNA763973.

## Ethics Statement

The studies involving human participants were reviewed and approved by Ethics Committee of the Shenzhen Center for Disease Control and Prevention. Written informed consent from the participants’ legal guardian/next of kin was not required to participate in this study in accordance with the national legislation and the institutional requirements.

## Author Contributions

LX analyzed the data and drafted the manuscript. BP collected the samples. LX, BP, YH, HC, XZ, MJ, LZ, QC, SW, YL and YQ performed the experiments. YC and YW contributed to data curation. XS, QH, and LC supervised the project. XS designed and revised the manuscript. LX and BP contributed equally to this work. All authors contributed to the article and approved the submitted version.

## Funding

This research was supported by the China National Science and Technology Major Projects Foundation (no. 2017ZX10303406), National Natural Science Foundation of China (no. 81773436), Sanming Project of Medicine in Shenzhen (no. SZSM201811071) and Shenzhen Key Medical Discipline Construction Fund (no. SZXK064).

## Conflict of Interest

The authors declare that the research was conducted in the absence of any commercial or financial relationships that could be construed as a potential conflict of interest.

## Publisher’s Note

All claims expressed in this article are solely those of the authors and do not necessarily represent those of their affiliated organizations, or those of the publisher, the editors and the reviewers. Any product that may be evaluated in this article, or claim that may be made by its manufacturer, is not guaranteed or endorsed by the publisher.

## Footnotes

^1^ http://bowtie-bio.sourceforge.net/bowtie2/index.shtml

^2^ https://github.com/biobakery/MetaPhlAn

^3^ https://github.com/SegataLab/hclust2

^4^ http://huttenhower.sph.harvard.edu/graphlan

^5^ https://github.com/tseemann/snippy

^6^ http://www.mgc.ac.cn/VFs/

^7^ https://cge.cbs.dtu.dk/services/ResFinder/

^8^ https://microbenet.cdc.gov
