# Supplementary material for: Isolation of Elizabethkingia anophelis From COVID-19 Swab Kits
Source: Front Microbiol. 2022 Jan 4;12:799150. doi: 10.3389/fmicb.2021.799150 (PMC8763855; doi:10.3389/fmicb.2021.799150)
Supplement: Supplementary file 1 [file Table_1.DOCX]

# Supplementary Tables

**Table S1.** **Sample collection information**

|  | Patient ID | age | Gender | Symptom | Manufacturer and batch of sampling kits | Sample ID | Metagenomic data SRA accession | Serum collection | Isolate ID | Isolate genome data SRA accession |
| --- | --- | --- | --- | --- | --- | --- | --- | --- | --- | --- |
| Fever cluster 1 (Middle School 1) | 1 | 15 | Female | Fever | Manufacturer 1, batch 1 | Sample 17 (Throat swab) | SRR16349056 | Yes | SZ17 | SRR15959140 |
|  | 2 | 15 | Male | Fever | Manufacturer 1, batch 1 | Sample 18 (Throat swab) | SRR16349057 | No | SZ18 | SRR15959139 |
|  | 3 | 15 | Female | Fever | Manufacturer 1, batch 1 | Sample 19 (Throat swab) | SRR16349058 | No | SZ19 | SRR15959128 |
|  | 4 | 14 | Female | Fever | Manufacturer 1, batch 1 | Sample 20 (Throat swab) | SRR16349059 | Yes | SZ20 | SRR15959117 |
|  | 5 | 14 | Female | Fever | Manufacturer 1, batch 1 | Sample 21(Throat swab) | SRR16349060 | Yes | SZ21 | SRR15959115 |
|  | 6 | 14 | Male | Fever | Manufacturer 1, batch 1 | Sample 22 (Throat swab) | SRR16349061 | Yes | SZ22 | SRR15959114 |
|  | 7 | 15 | Male | Fever | Manufacturer 1, batch 1 | Sample 23 (Throat swab) | SRR16349062 | Yes | SZ23 | SRR15959113 |
|  | 8 | 15 | Male | Fever | Manufacturer 1, batch 1 | Sample 24 (Throat swab) | SRR16349063 | Yes | SZ24 | SRR15959112 |
|  | 9 | 16 | Male | Fever | Manufacturer 1, batch 1 | Sample 25 (Throat swab) | SRR16349064 | Yes | SZ25 | SRR15959111 |
|  | 10 | 14 | Female | Fever | Manufacturer 1, batch 1 | Sample 26 (Throat swab) | SRR16349066 | Yes | SZ26 | SRR15959110 |
|  | 11 | 15 | Male | Fever | Manufacturer 1, batch 1 | Sample 27 (Throat swab) | SRR16349067 | Yes | SZ27 | SRR15959138 |
|  | 12 | 15 | Female | Fever | Manufacturer 1, batch 1 | Sample 28 (Throat swab) | SRR16349068 | Yes | SZ28 | SRR15959137 |
|  | 13 | 16 | Male | Fever | Manufacturer 1, batch 1 | Sample 29 (Throat swab) | SRR16349069 | Yes | SZ29 | SRR15959136 |
|  | 14 | 16 | Female | Fever | Manufacturer 1, batch 1 | Sample 30 (Throat swab) | SRR16349070 | Yes | SZ30 | SRR15959135 |
|  | 15 | 15 | Female | Fever | Manufacturer 1, batch 1 | Sample 449 (Throat swab) | SRR16349071 | Yes | SZ449 | SRR15959134 |
| Fever cluster 2 (Middle School 2) | 16 | 18 | Female | Fever | Manufacturer 1, batch 2 | Sample 5977 (Throat swab) | SRR16349072 | No | SZ5977 | SRR15959133 |
|  | 17 | 18 | Female | diarrhea | Manufacturer 1, batch 2 | Sample 5978 (Throat swab) | SRR16349073 | No | SZ5978 | SRR15959132 |
|  | 18 | 17 | Female | Fever | Manufacturer 1, batch 2 | Sample 5980 (Throat swab) | SRR16349074 | No | SZ5980 | SRR15959131 |
|  | 19 | 19 | Female | Fever and diarrhea | Manufacturer 1, batch 2 | Sample 5982 (Throat swab) | SRR16349075 | No | SZ5982 | SRR15959130 |
|  |  |  |  |  | Manufacturer 1, batch 2 | Sample 5983 (Anal swab) | SRR16349049 | No | SZ5983 | SRR15959129 |
|  | 20 | 18 | Female | Fever | Manufacturer 1, batch 2 | Sample 5984 (Throat swab) | SRR16349050 | No | SZ5984 | SRR15959127 |
|  |  |  |  |  | Manufacturer 1, batch 2 | Sample 5985 (Anal swab) | SRR16349051 | No | SZ5985 | SRR15959126 |
|  | 21 | 18 | Female | Fever | Manufacturer 1, batch 2 | Sample 5986 (Throat swab) | SRR16349052 | No | SZ5986 | SRR15959125 |
|  |  |  |  |  | Manufacturer 1, batch 2 | Sample 5987 (Anal swab) | SRR16349053 | No | SZ5987 | SRR15959124 |
| Fever cluster 3 (Middle School 3) | 22 | 19 | Male | Fever | Manufacturer 1, batch 3 | Sample 17325 (Throat swab) | - | No | SZ17325 | SRR15959123 |
|  | 23 | 19 | Male | Fever | Manufacturer 1, batch 3 | Sample 17327 (Throat swab) | - | No | SZ17327 | SRR15959122 |
|  | 24 | 19 | Male | Fever | Manufacturer 1, batch 3 | Sample 17329 (Throat swab) | - | No | SZ17329 | SRR15959121 |
|  | 25 | 18 | Male | Fever | Manufacturer 1, batch 3 | Sample 17330 (Throat swab) | - | No | SZ17330 | SRR15959120 |
|  | 26 | 18 | Male | Fever | Manufacturer 1, batch 3 | Sample 17331 (Throat swab) | - | No | SZ17331 | SRR15959119 |
|  | 27 | 18 | Male | Fever | Manufacturer 1, batch 3 | Sample 17332 (Throat swab) | - | No | SZ17332 | SRR15959118 |
|  | 28 | 17 | Female | Fever | Manufacturer 1, batch 3 | Sample 17333 (Throat swab) | - | No | SZ17333 | SRR15959116 |
| Blank-samples | - | - | - | - | Manufacturer 1, batch 3 | Blank-sample 1 | SRR16349054 | - | - | - |
|  | - | - | - | - | Manufacturer 1, batch 3 | Blank-sample 2 | SRR16349055 | - | - | - |
|  | - | - | - | - | Manufacturer 1, batch 3 | Blank-sample 3 | SRR16349065 | - | - | - |
|  | - | - | - | - | Manufacturer 1, batch 3 | Blank-sample 4 | SRR16349076 | - | - | - |
|  | - | - | - | - | Manufacturer 1, batch 3 | Blank-sample 5 | SRR16349077 | - | - | - |
|  | - | - | - | - | Manufacturer 1, batch 3 | Blank-sample 6-35 | - | - | - | - |
| Control samples | - | - | - | - | Manufacturer 2 | Control sample 1-13 (13 in total) | - | - | - | - |
|  | - | - | - | - | Manufacturer 3 | Control sample 14-22 (9 in total) | - | - | - | - |

**Table S2. Results of 47 biochemical reaction tests**

| Ala-Phe-Pro-ARYLAMIDASE | **+** | ADONITOL | **－** |
| --- | --- | --- | --- |
| L-ARABITOL | **－** | D-CELLOBIOSE | **－** |
| H2S PRODUCTION | **－** | BETA-N-ACETYL-GLUCOSAMINIDASE | **+** |
| D-GLUCOSE | **－** | GAMMA-GLUTAMYL-TRANSFERASE | **+** |
| BETA-GLUCOSIDASE | **+** | D-MALTOSE | **－** |
| D-MANNOSE | **±** | BETA-XYLOSIDASE | **－** |
| L-Proline ARYLAMIDASE | **+** | LIPASE | **±** |
| Tyrosine ARYLAMIDASE | **+** | UREASE | **－** |
| SACCHAROSE/SUCROSE | **－** | D-TAGATOSE | **－** |
| CITRATE(SODIUM) | **－** | MALONATE | **－** |
| L-LACTATE alkalinisation | **－** | ALPHA-GLUCOSIDASE | **+** |
| Beta-N-ACETYL-GALACTOSAMINIDASE | **+** | ALPHA-GALACTOSIDASE | **+** |
| Glycine ARYLAMIDASE | **+** | ORNITHINE DECARBOXYLASE | **－** |
| L-HISTIDINE assimilation | **－** | COURMARATE | **－** |
| O/129 RESISTANCE (comp.vibrio.) | **－** | Glu-Gly-Arg-ARYLAMIDASE | **+** |
| ELLMAN | **－** | L-LACTATE assimilation | **－** |
| L-Pyrrolydonyl-ARYLAMIDASE | **+** | D-TREHALOSE | **－** |
| BETA-GALACTOSIDASE | **±** | 5-KETO-D-GLUCONATE | **－** |
| Glutamyl Arylamidase pNA | **+** | SUCCINATE alkalinisation | **－** |
| FERMENTATION/ GLUCOSE | **－** | PHOSPHATASE | **+** |
| D-MANNITOL | **－** | LYSINE DECARBOXYLASE | **－** |
| BETA-Alanine arylamidase pNA | **－** | BETA-GLUCORONIDASE | **－** |
| PALATINOSE | **－** | L-MALATE assimilation | **－** |
| D-SORBITOL | **－** |  |  |
